# Safety and efficacy of a genetic vaccine targeting Telomerase plus chemotherapy for the therapy of canine B-cell lymphoma

**DOI:** 10.1186/2051-1426-1-S1-P212

**Published:** 2013-11-07

**Authors:** Alessandra Gavazza, George Lubas, Arthur Fridman, Daniela Peruzzi, Joseph A Impellizeri, Laura Luberto, Emanuele Marra, Giuseppe Roscilli, Gennaro Ciliberto, Luigi Aurisicchio

**Affiliations:** 1 University of Pisa, Pisa, Italy; 2 Merck & Co., West Point, NJ, USA; 3 University La Sapienza, Rome, Italy; 4 VSCHV, Wappinger Falls, NY, USA; 5 Takis, Rome, Italy; 6 IRCSS Pascale, Naples, Italy

## 

Client-owned pet dogs represent exceptional translational models for advancement of Cancer Research, as they reflects the complex heterogeneity observed in human cancer. We have recently shown that a genetic vaccine targeting dog telomerase (dTERT) and based on Ad/DNA-EGT technology can induce strong cell-mediated immune responses against this tumor antigen and increase overall survival of dogs affected by B-cell lymphosarcoma (LSA) in comparison with historical controls when combined with COP chemotherapy regimen. Here, we have conducted a double arm clinical trial with an extended number of LSA patients, measured the antigen-specific immune response and evaluated potential toxic effects of the immunotherapy along with a follow up of patients survival for three years and half. The immune response was measured by ELISPOT. The expression of dTERT was quantified by quantitative PCR. Changes in hematological parameters, local/systemic toxicity or organic dysfunction and fever were monitored over time during the treatment. dTERT-specific cell mediated immune responses were induced in almost all treated animals. No adverse effects were observed in any dog patient that underwent treatment. The overall survival time of vaccine/COP treated dogs was significantly increased over the COP-only treated cohort (>76.1 vs 29.3 weeks, respectively, p<0.0001). There was a significant association between dTERT expression levels in LSA cells and overall survival (OS) among vaccinated patients. In conclusion, Ad/DNA-EGT-based cancer vaccine against dTERT in combination with COP chemotherapy is safe and significantly prolongs the survival of LSA canine patients. These data confirm the therapeutic efficacy of dTERT vaccine and support the evaluation of this approach for other cancer types as well as the translation of this approach to human clinical trials.

